# Cutaneous vasculitis as a side effect of olaparib

**DOI:** 10.1016/j.jdcr.2025.06.043

**Published:** 2025-07-17

**Authors:** Paula Andrea Ramos Chaparro, Camila Andrea Zambrano Solorzano, Mariam Rolón Cadena, Maria Liliana Marino

**Affiliations:** a Primary Care Physician, Universidad de La Sabana, Chía, Colombia; b Dermatopathologist, Pathology Department, Fundación Santa Fe de Bogotá, Bogotá, Colombia; c Dermatologist, Dermatology Department, Fundación Santa Fe de Bogotá, Bogotá, Colombia

**Keywords:** drug response, medical dermatology, oncology, ovarian cancer, vasculitis

## Introduction

Olaparib, a polymerase adenosine diphosphate-ribose inhibitor, is the treatment that is currently approved for maintenance in women with platinum-sensitive recurrent ovarian cancer, regardless of breast cancer gene mutation status. It is also approved for advanced ovarian cancer in women with a deleterious germline breast cancer gene mutation who have received three or more lines of chemotherapy, irrespective of their response to platinum therapy.^1^ In a randomized, double-blind study, olaparib was found to reduce the risk of disease progression or death by 70% compared to placebo, demonstrating the drug’s efficacy.^1^

Adverse effects of olaparib frequently reported include decreased white blood cell count, anemia, nausea, diarrhea, and arthralgia.^2^ The most common skin reactions induced by olaparib are pruritus, alopecia, and erythematous rash,^3^ with cutaneous vasculitis reported in only one case, in an 80-year-old Japanese woman with recurrent stage IVB ovarian cancer. During treatment with olaparib (40 mg/day), the patient developed multiple purpuric lesions with subcutaneous induration on the lower left leg. Histopathological examination revealed dense perivascular infiltration of lymphocytes and neutrophils and fibrinoid necrosis of vessel walls in small arteries.^4^ Vasculitis involves vessel wall inflammation and is categorized by vessel size. Small vessel vasculitis is usually caused by drugs.^5^

## Case report

**Fig 1:**
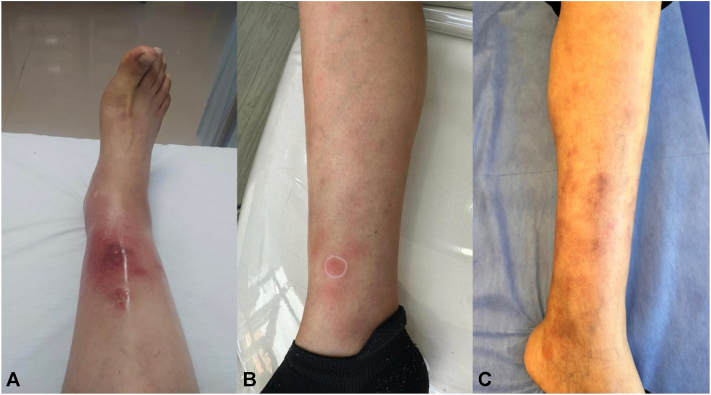
**A,** Physical findings at the first consultation: Erythematous nodules were observed on the lower limbs, which were painful on palpation. **B,** Physical findings at the second consultation: Erythematous nodules were observed on the lower limbs, with significant improvement compared to the previous consultation. **C,** Physical findings at the third consultation: In the lower limbs it finds erythematous nodules on the feet and violaceous macules that give way to acupressure with a residual appearance of edema without fovea and pain on palpation.

This report presents the case of a patient who developed vasculitis of small- and medium-sized vessels after maintenance treatment with olaparib for metastatic ovarian cancer. The patient, who presented with swelling and pain in the right lower limb after starting therapy with olaparib at a dose of 300 mg every 12 hours, initially attributed these symptoms to an insect bite (Fig 1).

**Fig 2:**
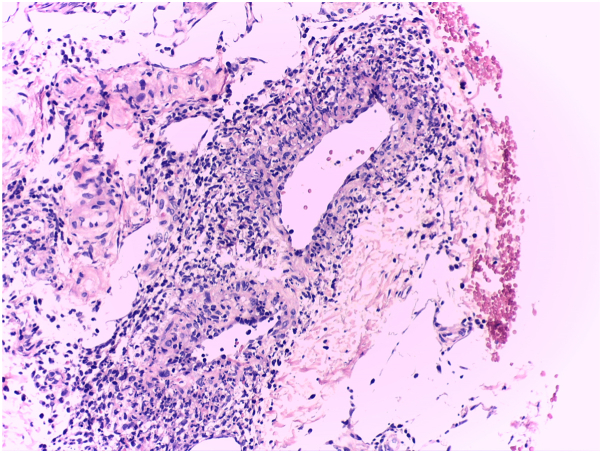
Photomicrograph with 20× magnification in hematoxylin eosin stain showing severe vascular involvement with mononuclear inflammation surrounding and permeating medium- and small-sized vascular structures, along with evidence of nuclear fragmentation (karyorrhexis).

Subsequent investigations, such as a Doppler ultrasound, ruled out the presence of thrombophlebitis, and the temporary interruption of olaparib produced a significant improvement, after which maintenance therapy with olaparib was restarted at a dose of 150 mg every 12 hours, with acceptable tolerance. Biopsy for hematoxylin and eosin staining revealed a mononuclear infiltration in small- and medium-caliber blood vessels overall consistent with a leukocytoclastic vasculitis (Fig 2). Direct immunofluorescence was not required.

Additional workup to identify extracutaneous manifestations of vasculitis was performed, including antineutrophil cytoplasmic antibodies. The results of the laboratory tests showed the following values: rheumatoid factor less than 15, antistreptolysin less than 50, C3 level at 109, C4 level at 19.9, negative results for extractable nuclear antigen tests, antineutrophil cytoplasmic antibodies, and antinuclear antibodies at 1:320 with a fine granular pattern, and negative cryoglobulins.

In the context of the above negative workup for a primary rheumatologic cause of the patient's small and medium vessel cutaneous vasculitis, the patient's olaparib was deemed the most likely driver of her cutaneous symptoms. Five months after the first dermatology consultation, the patient returned to the clinic due to the reappearance of erythematous nodules on the lower limbs (Fig 1), after increasing the dose of olaparib to 300 mg every 12 hours, due to positron emission tomography scan findings of malignant hypermetabolic lymph nodes and elevated cancer antigen 125 antigen, reaffirming the side effect of the medication. The patient benefited from symptomatic management, which included dose adjustment, a short course of methylprednisolone, and topical treatment with corticosteroids and tacrolimus.

Upon applying the Naranjo algorithm to assess the causality of an adverse drug reaction, a total of 4 points were obtained. These points were attributed to the occurrence of the adverse reaction following the administration of the drug, improvement observed upon discontinuation of the drug, and subsequent amelioration upon dose reduction.

## Discussion

Ovarian cancer is a serious and recurrent condition often characterized by impaired deoxyribonucleic acid repair mechanisms. Olaparib, a polymerase adenosine diphosphate-ribose inhibitor, is particularly effective in targeting breast cancer gene 1/2-mutated cancers by disrupting deoxyribonucleic acid repair, which induces cancer cell death and enhances the efficacy of chemotherapy. It is also recognized as a maintenance treatment for patients with platinum-sensitive recurrent ovarian cancer.^1^

Although generally is well-tolerated as a standalone treatment with mild side effects like fatigue, nausea, and vomiting, combining olaparib with cytotoxic chemotherapy may heighten toxicity, necessitating dosage adjustments.^6^ There are several olaparib-induced skin disorders, including pruritus, alopecia, and erythematous rash.^6^

Ovarian carcinoma belongs to the group of tumors considered high risk for developing venous thromboembolism or deep vein thrombosis, with highly variable incidence rates (5% to 20%).^6^ In our case, once deep vein thrombosis was ruled out, a consultation was requested from the dermatology service, who diagnosed vasculitis, after a clinical evaluation, where it was evidenced (Fig 1) and a skin biopsy, which was evidenced (Fig 2).

Cutaneous small-vessel vasculitis is typically characterized by palpable purpura resulting from leukocytoclastic inflammation of postcapillary venules. It may be idiopathic or triggered by drugs, infections, or systemic diseases.^5^ In our case, the patient presented with painful nodules on the pretibial surface of the right lower limb, resembling erythema nodosum. However, the biopsy result revealed mononuclear infiltration in small- and medium-sized vessels (Fig 2), confirming the diagnosis of vasculitis.

Managing vasculitis involves discontinuing the triggering drug, although many cases require immunosuppressive therapy. Our patient responded well to a reduced dose of olaparib and topical corticosteroids, avoiding the need for additional therapies. Permanent discontinuation of olaparib was not considered due to its significant efficacy in treating ovarian cancer.

## Conclusion

This case report discusses a rare adverse effect of olaparib, vasculitis, in a stage IV ovarian cancer patient. While olaparib is effective, it can cause complications requiring careful management. The patient’s successful symptomatic treatment highlights the need for clinicians to be aware of this rare side effect to ensure effective and safe cancer care.

## Conflicts of interest

None disclosed.
